# Feel4Diabetes healthy diet score: development and evaluation of clinical validity

**DOI:** 10.1186/s12902-020-0521-x

**Published:** 2020-05-06

**Authors:** Eeva Virtanen, Jemina Kivelä, Katja Wikström, Christina-Paulina Lambrinou, Pilar De Miguel-Etayo, Nele Huys, Katalin Vraukó-Tóth, Luis A. Moreno, Natalya Usheva, Nevena Chakarova, Sándorné A. Rado, Violeta Iotova, Konstantinos Makrilakis, Greet Cardon, Stavros Liatis, Yannis Manios, Jaana Lindström, on behalf of the Feel4Diabetes research group, Yannis Manios, Greet Cardon, Jaana Lindström, Peter Schwarz, Konstantinos Makrilakis, Lieven Annemans, Ignacio Garamendi, Kalliopi Karatzi, Odysseas Androutsos, George Moschonis, Spyridon Kanellakis, Christina Mavrogianni, Konstantina Tsoutsoulopoulou, Christina Katsarou, Eva Karaglani, Irini Qira, Efstathios Skoufas, Konstantina Maragkopoulou, Antigone Tsiafitsa, Irini Sotiropoulou, Michalis Tsolakos, Effie Argyri, Mary Nikolaou, Eleni-Anna Vampouli, Christina Filippou, Kyriaki Apergi, Amalia Filippou, Gatsiou Katerina, Efstratios Dimitriadis, Tiina Laatikainen, Katja Wikström, Jemina Kivelä, Päivi Valve, Esko Levälahti, Eeva Virtanen, Tiina Pennanen, Seija Olli, Karoliina Nelimarkka, Vicky Van Stappen, Nele Huys, Ruben Willems, Samyah Shadid, Patrick Timpel, Stavros Liatis, George Dafoulas, Christina-Paulina Lambrinou, Angeliki Giannopoulou, Lala Rabemananjara, Maria Stella de Sabata, Winne Ko, Luis Moreno, Fernando Civeira, Gloria Bueno, Pilar De Miguel-Etayo, Esther Mª. Gonzalez-Gil, María L. Miguel-Berges, Natalia Giménez-Legarre, Paloma Flores-Barrantes, Aleli M. Ayala-Marín, Miguel Seral-Cortés, Lucia Baila-Rueda, Ana Cenarro, Estíbaliz Jarauta, Rocío Mateo-Gallego, Violeta Iotova, Tsvetalina Tankova, Natalia Usheva, Kaloyan Tsochev, Nevena Chakarova, Sonya Galcheva, Rumyana Dimova, Yana Bocheva, Zhaneta Radkova, Vanya Marinova, Yuliya Bazdarska, Tanya Stefanova, Imre Rurik, Timea Ungvari, Zoltán Jancsó, Anna Nánási, László Kolozsvári, Csilla Semánova, Éva Bíró, Emese Antal, Sándorné Radó, Remberto Martinez, Marcos Tong

**Affiliations:** 1 Department of Public Health Solutions, National Institute for Health and Welfare, PO BOX 30, 00270 Helsinki, Finland; 2 Department of Obstetrics and Gynecology, University of Helsinki and Helsinki University Hospital, Helsinki, Finland; 3 Institute of Public Health and Clinical Nutrition, University of Eastern Finland, Kuopio, Finland; 4 Department of Nutrition and Dietetics, Harokopio University, Athens, Greece; 5 Growth, Exercise, Nutrition and Development Research Group, University of Zaragoza, GENUD, Zaragoza, Spain; 6 Department of Movement and Sports Sciences, Ghent University, Ghent, Belgium; 7 Debreceni Egyetem (UoD), University of Debrecen, Debrecen, Hungary; 8 Department of Social Sciences and Public Health, Medical University of Varna, Varna, Bulgaria; 9 Department of Diabetology, Clinical Center of Endocrinology, Medical University of Sofia, Sofia, Bulgaria; 10 Department of Pediatrics, Clinic of Paediatric Endocrinology, Medical University Varna, Varna, Bulgaria; 11 National and Kapodistrian University of Athens, Athens, Greece

**Keywords:** Diet, Diet score, Evaluation, Intervention, Risk factors, Type 2 diabetes, Validity

## Abstract

**Background:**

The aim of this paper is to present the development of the Feel4Diabetes Healthy Diet Score and to evaluate its clinical validity.

**Methods:**

Study population consisted of 3268 adults (63% women) from high diabetes risk families living in 6 European countries. Participants filled in questionnaires at baseline and after 1 year, reflecting the dietary goals of the Feel4Diabetes intervention. Based on these questions the Healthy Diet Score was constructed, consisting of the following components: breakfast, vegetables, fruit and berries, sugary drinks, whole-grain cereals, nuts and seeds, low-fat dairy products, oils and fats, red meat, sweet snacks, salty snacks, and family meals. Maximum score for each component was set based on its estimated relative importance regarding T2DM risk, higher score indicating better quality of diet. Clinical measurements included height, weight, waist circumference, heart rate, blood pressure, and fasting blood sampling, with analyses of glucose, total cholesterol, HDL-cholesterol, LDL-cholesterol, and triglycerides.

Analysis of (co) variance was used to compare the Healthy Diet Score and its components between countries and sexes using baseline data, and to test differences in clinical characteristics between score categories, adjusted for age, sex and country. Pearson’s correlations were used to study the association between changes from baseline to year 1 in the Healthy Diet Score and clinical markers. To estimate reproducibility, Pearson’s correlations were studied between baseline and 1 year score, within the control group only.

**Results:**

The mean total score was 52.8 ± 12.8 among women and 46.6 ± 12.8 among men (*p* <  0.001). The total score and its components differed between countries. The change in the Healthy Diet Score was significantly correlated with changes in BMI, waist circumference, and total and LDL cholesterol. The Healthy Diet Score as well as its components at baseline were significantly correlated with the values at year 1, in the control group participants.

**Conclusion:**

The Feel4Diabetes Healthy Diet Score is a reproducible method to capture the dietary information collected with the Feel4Diabetes questionnaire and measure the level of and changes in the adherence to the dietary goals of the intervention. It gives a simple parameter that associates with clinical risk factors in a meaningful manner.

**Trial registration:**

Clinicaltrials.gov NCT02393872. Registered March 20, 2015.

## Background

Type 2 diabetes (T2DM) is a chronic disease that develops as a result of interactions between genetic and environmental factors. In addition to obesity and sedentary lifestyle, the composition of the diet is recognized as one of the important modifiable factors in the development of T2DM [1]. Lifestyle interventions focusing on dietary modification and increasing physical activity have been shown to prevent the development of T2DM in people at high risk [2]. To evaluate the effectiveness of a preventive intervention, it is thus important to be able to measure the achieved changes in lifestyle using validated methods.

One of the aims in the multinational Feel4Diabetes project was to identify families at increased T2DM risk and provide them interventions to decrease the risk in a cluster-randomized study setting in 6 European countries (Belgium, Finland, Greece, Spain, Hungary, Bulgaria) [3]. The high-risk families living in the intervention areas were subject to the interventions delivered in the school and community setting between 2016 and 2018. In addition, they were offered a possibility to take part in a more intensive lifestyle counselling, consisting of group and individual sessions and SMS-intervention focusing specifically on preventing the development of T2DM.

To measure the effect of the Feel4Diabetes intervention on dietary behaviours, questions on dietary intake were included in the questionnaire which the parents filled in at baseline and after 1 and 2 years [3, 4]. The dietary questions were formulated to focus specifically on the intake of the food items that had been chosen as the goals in the Feel4Diabetes dietary intervention. To test the questionnaire reliability, a total of 191 parents completed the questionnaires twice, with a 1–2 week interval, and based on the intra-class correlation coefficients (ICC) of test-retest the developed questionnaires showed acceptable reliability [4]. Based on the dietary questions, the Feel4Diabetes Healthy Diet Score was compiled, in order to simplify the information from the questionnaire in a single parameter that can be used as a marker of dietary compliance.

The aim of this paper is to present the development of the Feel4Diabetes Healthy Diet Score and to evaluate its clinical validity.

## Methods

### Study population and measurements

Participants were 3268 adults (63% women) from high diabetes risk families living in the Feel4Diabetes study areas in 6 European countries. A high-risk family was defined as at least one of the parents having (relatively) high score in the Finnish Diabetes Risk Score FINDRISC [5]. FINDRISC is a validated tool to identify people at risk of developing T2DM in the future, consisting of eight questions (age, body mass index (BMI), waist circumference, physical activity, consumption of fruit and vegetables, blood pressure medication, high blood glucose measured at any point, and family history of diabetes). The FINDRISC Score ranges from 0 to 26, with score 12 or higher indicating increased T2DM risk. The mean age of the study participants was 42 ± 7 years, and 82% of them were married or lived with a spouse. The average years of education completed were 14–15 years, and 67% of the participants worked full or part-time during the intervention period. The recruitment of the participants, as well as the procedures and measurements, has been explained in detail earlier [3]. In brief, the adults who consented to take part in the clinical study were invited to a study visit. The measurements included height, weight, waist circumference, heart rate, blood pressure, and fasting blood sampling, with analyses of glucose, total cholesterol, HDL-cholesterol, LDL-cholesterol, and triglycerides. Body mass index (BMI) was calculated as weight (kg) divided by height (m) squared. Waist-to-height ratio (WHtR) was calculated dividing waist circumference (m) by height (m). Participants also filled in a questionnaire including dietary questions, which were used to develop components of the Score (see Additional file 1). The measurements were repeated after 1 year.

### Feel4Diabetes healthy diet score

Dietary goals set in Feel4Diabetes intervention were used as the basis for the Feel4Diabetes Healthy Diet Score. There were a total of 12 Feel4Diabetes intervention goals that were related to food choices or food behaviour, and were selected as the main components of the Healthy Diet Score (Table 1). These components were breakfast, vegetables, fruit and berries, sugary drinks, whole-grain cereals, nuts and seeds, low-fat dairy products, oils and fats, red meat, sweet snacks, salty snacks, and family meals. Excluded intervention goals were related to physical activity and sedentary behaviour, and thus not included as part of the Healthy Diet Score.

**Table 1:** Scoring of the Healthy Diet Score

Component and F4D dietary goal	Content	Categories	Score
1. Breakfast Eating (healthy and balanced) breakfast daily	Weekdays and weekend days	5 times on weekdays	7
		3–4 times on weekdays	4
		1–2 times on weekdays	1
		I don’t eat breakfast	0
		2 times during weekend	3
		1 time during weekend	2
		I don’t eat breakfast	0
		Total score	10
2. Vegetables ≥ 5 servings of vegetable per day	Raw and cooked vegetables, cooked legumes	5 servings per day	10
		4 servings per day	8
		3 servings per day	6
		2 servings per day	4
		1 servings per day	2
		< 1 serving per day	0
3. Fruits and berries ≥ 3 servings of fruit & berries per day	Fruit and berries	3 or more servings per day	10
		1–2 servings per day	8
		5–6 servings per week	6
		3–4 servings per week	4
		1–2 servings per week	2
		< 1 serving per week	0
4. Sugary drinks < 1 serving of sugary drinks per day	Soft drinks with sugar, juices with sugar, beer, cider, wine, spirits	< 1 serving per day	10
		1–2 servings per day	8
		3–4 servings per day	6
		5–6 servings per day	4
		≥ 7 servings per day	0
5. Whole-grain cereals ≥ 4 servings of whole-grain foods/cereals per day	Whole-grain bread, porridge, whole-grain cereals	≥ 4 servings per day	10
		3 servings per day	7
		2 servings per day	4
		1 serving per day	1
		< 1 serving per day	0
6. Nuts and Seeds ≥ 3 servings of nuts & seeds a week	Nuts and seeds	≥ 3 servings per week	6
		1–2 servings per week	3
		< 1 time per week	0
7. Low-fat Dairy ≥ 1 serving of low-fat dairy products per day	Low-fat dairy < 2% fat Full-fat dairy > 2% fat	≥ 1 servings per day and no full-fat dairy products	6
		≥ 1 servings per day and ≥ 1 servings of full-fat dairy products	3
		< 1 serving per day and ≤ 1 servings of full-fat dairy products	0
8. Oils and fats daily use of olive or rapeseed oil or soft margarine including cooking fats and spreads	Favorable fats: vegetable oils, margarine, soft and reduced-fat margarine, oil based dressings Avoidable fats: butter, butter mixtures, I don’t use any	Cooking (option to choose several):	
		Daily use of vegetable oils and margarines and no use of butter	4
		Daily use of vegetable oils and margarines and some usage of butter	2
		Daily use or no usage of vegetable oils or margarines and higher usage of butter or no usage of daily fats	0
		Bread spreads (option to choose only one):	4
		Daily use of vegetable oil, Soft margarine 70–80% or fat	0
		Reduced-fat margarine 28–60% fat	
		Daily use of butter-vegetable oil mixture, butter or I do not use fat spread on bread	8
		Total score	
9. Red meat ≤ 2 servings of red and/or processed meat a week	Red and processed meat	≤ 2 servings per week	10
		3 servings per week	8
		4 servings per week	6
		5 servings per week	4
		6 servings per week	2
		≥ 7 servings per week	0
10. Sweet snacks ≤ 1 serving of sweet snacks per week	Biscuits, ice cream, cakes, pastries etc.	≤ 1 servings per week	6
		2 servings per week	5
		3–4 servings per week	3
		5–6 servings per week	1
		≥1 servings per day	0
11. Salty snacks ≤ 1 serving of salty snacks/fast food per week	Hamburger, chips, pizza, savory pastries etc.	≤ 1 servings per week	6
		2 servings per week	5
		3–4 servings per week	3
		5–6 servings per week	1
		≥1 servings per day	0
12. Family meals Meals eaten with others	Breakfast, lunch and dinner with a friend, colleague, or with a family member	Breakfast	
		≥ 7 times per week	2
		5–6 times per week	1
		≤ 4 times per week	0
		Lunch	
		≥ 7 times per week	2
		5–6 times per week	1
		≤ 4 times per week	0
		Dinner	
		≥ 7 times per week	4
		5–6 times per week	2
		≤ 4 times per week	0
		Total score	0–8
Total Healthy Diet Score	All components (1–12)		0–100

Scoring of the components was done according to the 14 diet-related questions in the Feel4Diabetes questionnaire. Each component reflected one or two questions about intake frequencies of each particular food group or food behaviour. Components consisting of two questions were vegetables and oils and fats. Vegetables included questions about vegetable and legume consumption, and oils and fats included questions about bread spreads and daily use of different oils and fats. The maximum score for each component was set based on its estimated relative importance with regards to T2DM risk. A maximum score of 10 was given to breakfast, vegetables, fruit and berries, sugary drinks (including juices and soft drinks containing sugar and also high energy beverages beer, cider, wine and spirits), whole-grain cereals, and red meat. A maximum score of 8 was given to the consumption of oils and fats and frequency of family meals (including breakfast, lunch and dinner eaten in the company of others, defined as a friend, colleague or family member). The rest of the components, sweet snacks, salty snacks, nuts and seeds, and low-fat dairy, got a maximum score of 6. A higher score indicated higher or more frequent consumption, except for sugary drinks, red meat, sweet snacks and salty snacks where higher scores indicated lower consumption. Total score, calculated as the sum of the component scores, was ranging from 0 to 100, higher score indicating better quality of diet and maximum score indicating full achievement of the Feel4Diabetes dietary goals.

### Descriptive and statistical analyses

Data was analyzed with IBM SPSS Statistics for Windows Version 25.0. The Healthy Diet Score components were calculated for each country and men and women separately using baseline data. Score component values, as country means for men and women, are presented as bar charts. The Feel4Diabetes Healthy Diet Score was divided into quarters. A multivariate analysis of covariance was used to test differences in clinical characteristics between the Score categories. In the model, clinical characteristics were used as dependent variables and score categories as the independent variable. The model was adjusted for age, sex and country and results are presented as marginal estimated means, standard deviations, and *p*-values.

To estimate the clinical validity of the Feel4Diabetes Healthy Diet Score, the changes in the Score and changes in clinical markers between baseline and first year follow-up were studied using Pearson’s correlation. To estimate reproducibility, Pearson’s correlations were studied between baseline and first year follow-up scores within the control group participants only. Results from both analyses using Pearson’s correlation are presented as correlation coefficients and *p*-values.

## Results

**Fig. 1:**
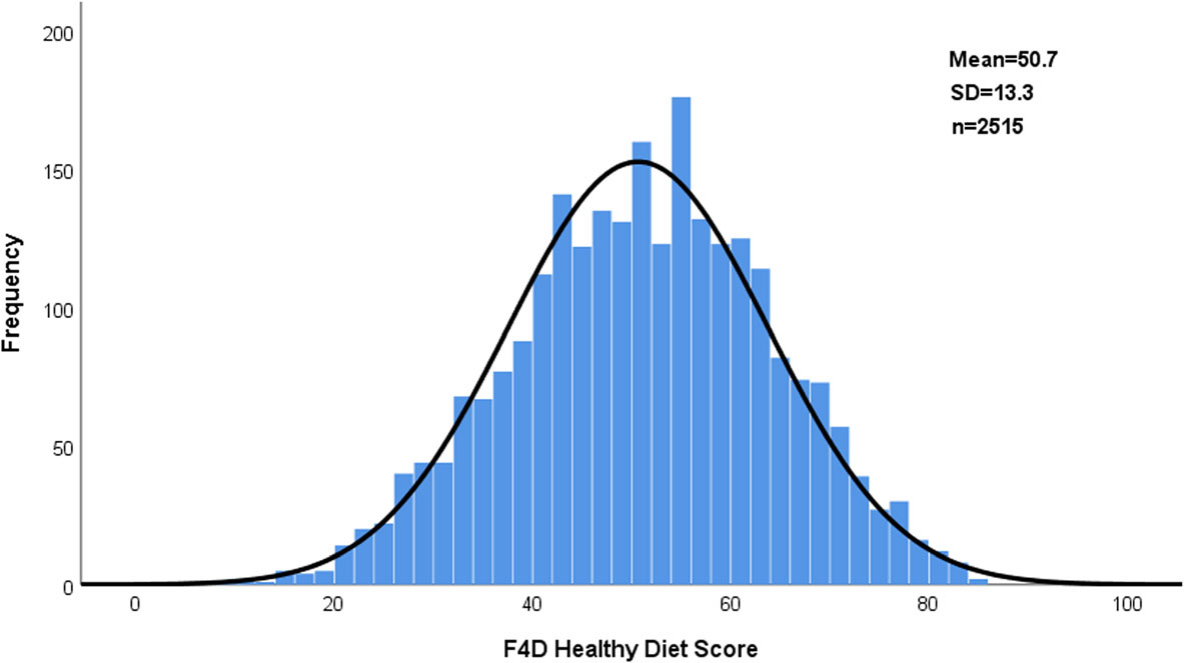
Distribution of the Healthy Diet Score in study population

The distribution of the Healthy Diet Score in the total population at the baseline is depicted in Fig. 1.

**Fig. 2:**
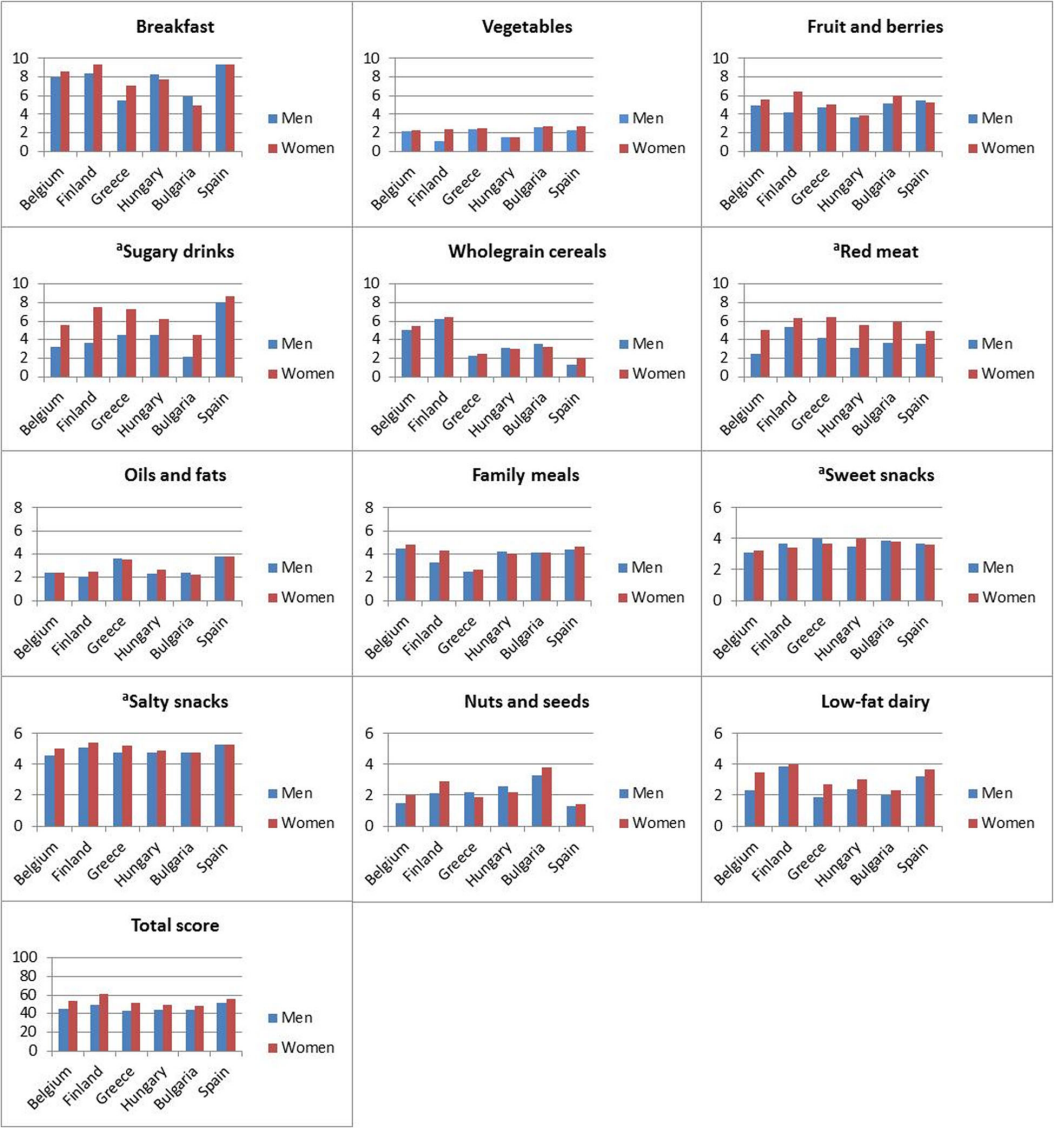
Feel4Diabetes Healthy Diet Score components by country and sex for all participants. ^a^ higher score indicating lower consumption

The mean total score was 52.8 ± 12.8 among women and 46.6 ± 12.8 among men (*p* <  0.001). The mean score values by sex and country are presented in Fig. 2. There were significant differences in the total score, as well as its components, between countries.

The total score was highest among Finnish (56.8 ± 11.9) and Spanish (52.9 ± 11.4), and lowest in Hungarian (47.8 ± 12.7) and Bulgarian (47.9 ± 11.5) adults. Of the components, score value was generally low for vegetables and high for salty snacks and breakfast.

The baseline clinical characteristics of all study participants according to Healthy Diet Score, adjusted for age, sex and country are presented in Table 2. The Score was directly associated with HDL-cholesterol (*p* = 0.022), and inversely associated with LDL-cholesterol (*p* = 0.003) and triglyceride (*p* = 0.043) concentrations as well as heart rate (*p* = 0.012).

**Table 2:** Baseline clinical characteristics of all participants by Healthy Diet Score quarters (EMM, SE and ANCOVA *p*-value)

	1. quarter 0–42	2. quarter 43–51	3. quarter 52–60	4. quarter 61–100	*p*-value
BMI (Kg/m^2^)	28.9 ± 0.2	28.7 ± 0.2	28.1 ± 0.2	28.2 ± 0.2	0.054
Waist circumference (cm)	95.3 ± 0.5	95.0 ± 0.6	94.0 ± 0.5	94.4 ± 0.6	0.325
WHtR	0.57 ± 0.0	0.57 ± 0.0	0.56 ± 0.0	0.56 ± 0.0	0.113
SBP (mmHg)	118 ± 1	119 ± 1	118 ± 1	119 ± 1	0.779
DBP (mmHg)	79 ± 1	79 ± 1	79 ± 1	79 ± 1	0.882
Heart rate (bpm)	73 ± 0	72 ± 0	72 ± 0	71 ± 0	0.012*
total Cholesterol (mmol/L)	5.12 ± 0.04	5.02 ± 0.04	5.03 ± 0.04	4.96 ± 0.04	0.058
LDL-cholesterol (mmol/L)	3.21 ± 0.04	3.10 ± 0.04	3.10 ± 0.04	3.02 ± 0.04	0.003*
HDL-cholesterol (mmolL)	1.36 ± 0.04	1.36 ± 0.04	1.38 ± 0.04	1.42 ± 0.04	0.022*
TG (mg/dL)	1.29 ± 0.04	1.31 ± 0.03	1.23 ± 0.03	1.17 ± 0.04	0.043*
Glucose (mmol/L)	5.24 ± 0.04	5.33 ± 0.05	5.31 ± 0.05	5.37 ± 0.05	0.240

*p <  0.05, ANCOVA adjusted for age, sex and country. *BMI* Body mass index, *WHtR* Waist-to-Height ratio, *SBP* Systolic blood pressure, *DBP* Diastolic blood pressure, *TG* Triglycerides, *EMM* Estimated marginal means, *SE* Standard error

The change in Healthy Diet Score from baseline to year 1, amongst all participants, was significantly and inversely correlated with changes in BMI, waist circumference, waist-to-height ratio and total and LDL cholesterol (Table 3). Increase in component scores for vegetables, fruit and berries, sweet snacks, salty snacks, and red meat correlated with a decrease of body weight indices, and increased intake of vegetables also with a decrease of total cholesterol and triglycerides. Higher consumption of whole-grains correlated only with a decrease of total and LDL-cholesterol. Improvement in breakfast habits correlated with a reduction in BMI, but improvement in family meal practices did not correlate with any clinical changes. Sugary drinks correlated only with a change in systolic blood pressure, but the correlation was not in the expected direction. Also for the change in sweet and salty snacks score components an inverse correlation with HDL-cholesterol was discovered.

**Table 3:** Pearson’s correlation for changes in the Healthy Diet Score components and clinical markers between baseline and first year for all participants

	BMI (kg/m^2^)	WC (cm)	WHtR	SBP (mmHg)	DBP (mmHg)	Heart rate (bpm)	TC (mmol/l)	LDL (mmol/l)	HDL (mmol/l)	Glucose (mmol/l)	TG (mmol/l)
Total score	− 0.143**	− 0.116**	− 0.123**	ns	ns	ns	−0.127**	− 0.114**	ns	ns	ns
Breakfast	−0.061*	ns	ns	ns	ns	ns	ns	ns	ns	ns	ns
Vegetables	−0.077**	−0.080**	−0.070**	ns	ns	ns	−0.052*	ns	ns	ns	−0.050*
Fruit and berries	−0.064*	−0.069**	− 0.061*	ns	ns	ns	ns	ns	ns	ns	ns
Sugary drinks	ns	ns	ns	0.053*	ns	ns	ns	ns	ns	ns	ns
Whole-grain	ns	ns	ns	ns	ns	ns	−0.065*	−0.062*	ns	ns	ns
Nuts and seeds	ns	−0.052*	−0.052*	ns	ns	ns	ns	ns	ns	ns	ns
Low-fat dairy	ns	ns	ns	ns	ns	ns	ns	ns	ns	ns	ns
Oils and fats	−0.052*	ns	ns	ns	ns	ns	ns	ns	ns	ns	ns
Red meat	−0.089**	−0.077**	− 0.078**	ns	ns	ns	ns	ns	ns	ns	ns
Sweet snacks	−0.120**	−0.098**	− 0.103**	ns	ns	ns	ns	ns	−0.061*	ns	ns
Salty snacks	−0.061*	−0.073**	− 0.070**	ns	ns	ns	ns	ns	−0.058*	ns	ns
Family meals	ns	ns	ns	ns	ns	ns	ns	ns	ns	ns	ns

*p <  0.05 and **p <  0.01. *ns* non-significant

*BMI* Body mass index, *WC* Waist circumference, *WHtR* Waist-to-Height ratio, *SBP* Systolic blood pressure, *DBP* Diastolic blood pressure, *TC* total cholesterol, *LDL* LDL-cholesterol, *HDL* HDL-cholesterol, *TG* Triglycerides

The Healthy Diet Score, as well as its components at baseline, were significantly correlated with the values at year 1 in the analysis including only the control group participants (Table 4). The Correlation coefficients varied from 0.479 (*p* <  0.001) for fats and oils to 0.795 (*p* <  0.001) for breakfast. For the total score, the correlation coefficient between baseline and year 1 was 0.755 (*p* <  0.001).

**Table 4:** Correlation for the Healthy Diet Score components between baseline and first year for controls

Score component	Pearson’s correlation	*p*-value
Total Score	0.755**	< 0.001
Breakfast	0.795**	< 0.001
Vegetables	0.486**	< 0.001
Fruit and berries	0.704**	< 0.001
Sugary drinks	0.622**	< 0.001
Whole-grain cereals	0.565**	< 0.001
Nuts and seeds	0.602**	< 0.001
Low-fat dairy	0.517**	< 0.001
Oils and fats	0.479**	< 0.001
Red meat	0.626**	< 0.001
Sweet snacks	0.624**	< 0.001
Salty snacks	0.558**	< 0.001
Family meals	0.616**	< 0.001

**p<0.01

## Discussion

In this paper, we report the construction of the Feel4Diabetes Healthy Diet Score and present some findings to support its usability, reproducibility and clinical validity. The Healthy Diet Score is constructed based on the questionnaire that was developed for the Feel4Diabetes project [4]. A detailed description of the study population and design, as well as study results, are out of the scope of this paper.

The Healthy Diet Score was created to standardize and simplify the Feel4Diabetes dietary questionnaire [4] data in a single parameter that allows rapid evaluation of the diet across this multi-national project. The Healthy Diet Score is comprised of components that represent the dietary goals of the Feel4Diabetes high-risk intervention, weighted based on their appraised importance as risk or protective factors for type 2 diabetes.

The weights of the components were decided based on research literature. The most convincing evidence about the potential of dietary manipulation in reducing T2DM risk comes from randomized prevention trials. The Finnish Diabetes Prevention Study (DPS) was the first individually-randomized controlled trial to show that T2DM can be prevented by dietary and physical activity counselling [6]. Risk of developing T2DM was shown to be the lowest among the participants who consumed a diet with moderate fat and high fibre content [7]. In the Diabetes Prevention Program, another important trial showing the effectiveness of lifestyle intervention in T2DM prevention, the diet was focused on the reduction of total fat and energy [8]. However, as we wanted the Feel4Diabetes Healthy Diet Score to be based on food rather than nutrient intake, research evidence from dietary patterns [1, 9] and other observational studies [10–15] were also considered when the scoring for the components was created.

Furthermore, we wanted the score to be sensitive for all beneficial changes in diet, e.g. increase in consumption of fruit from once per day to twice per day, even if the actual goal (three portions per day) is not achieved. This approach differs from the one used in many studies. For example in the DPS, the “success score” varied from 0 to 5, according to the number of intervention goals (total fat intake < 30% of total energy consumed, saturated fat < 10% of total energy, and dietary fibre 15 g/1000 kcal or more, physical activity 30 min per day, weight reduction 5% from baseline) [6]. Another example is the PREDIMED study, where the Mediterranean diet score was calculated as the sum of dichotomized goals [16]. In real-life implementation projects, achieved changes in diet are often smaller than in clinical trials. In fact, it is generally acknowledged that setting small, achievable short term goals, as well as focusing on gradually building on behaviours that already are familiar, increases the likelihood that the new way of eating becomes a permanent habit [17]. For the Feel4Diabetes Healthy Diet Score, we wanted to acknowledge all the changes in diet, even when the actual intervention goal was not achieved. The score was composed from all the relevant information available in the validated questionnaire, and all changes for the better increased the score.

The Healthy Diet Score at baseline was normally distributed and thus demonstrated variation within the study population. However, the country means of the Score, as well as its components, differed significantly from each other. These differences appeared plausible, considering the differing dietary cultures in the participating countries [18, 19]. For example, in Finland whole grain rye bread and low-fat milk are staples, and this is also reflected in the Score components for whole-grain cereal and dairy products. The consumption of vegetables was much lower than recommended across the countries, clearly indicating a need for public health actions to tackle the problem.

In the cross-sectional analysis, the Feel4Diabetes Healthy Diet Score category was significantly, albeit moderately associated with clinical risk factors such as HDL- and LDL-cholesterol and triglycerides, when age, sex, and country were adjusted for. There also appeared to be a trend between decreasing BMI and increasing score. This association did not reach statistical significance. In the analysis exploring the association between changes in the Score and its components as opposed to changes in clinical risk factors, several associations were discovered. These analyses combined offer proof for the clinical validity of our Healthy Diet Score. Many diet scores have been developed for different purposes, and they have been proved clinically useful, especially when combined with other non-invasive measurements of risk factors [20]. Lassale et al. who compared the performance of different scores advocate the use of easy-to-measure, concise food-based scores that are developed based on predefined rather than study population-based cut-off points and propose that such scores “would be most pragmatic for individual risk prediction and health promotion” [20]. This is indeed also our justification for the development of the Feel4Diabetes Healthy Diet Score.

Finally, the reproducibility of the Healthy Diet Score was shown to be good, based on the analysis of correlation between baseline and 1 year score components in the control participants only. This analysis excluded intervention subjects, as they were, by definition, instructed on how to make beneficial changes in their diet.

An important limitation of the present study is that we do not have a reference method (e.g. food diaries) for dietary data collection. However, the questionnaire reliability was tested in volunteers in each country before the study [4] and was shown to be acceptable.

## Conclusions

The Feel4Diabetes Healthy Diet Score is a reproducible method to capture the dietary information collected with the Feel4Diabetes questionnaire and measure the level of and changes in the adherence to the dietary goals of the intervention. It gives a simple parameter that associates with clinical risk factors in a meaningful and plausible manner. The Healthy Diet Score was constructed to facilitate the analyses of the Feel4Diabetes study outcomes, but it could also serve as a simple and visual tool to measure dietary intake in e.g. health care services and public health initiatives.

## Supplementary information

**Additional file 1.** Feel4Diabetes dietary questions used in the development of the Healthy Diet score.

## Data Availability

Not applicable.

## Abbreviations

BMI: Body mass index
DPS: Diabetes Prevention Study
FINDRISC: Finnish Diabetes Risk Score
HDL: High-density lipoprotein
LDL: Low-density lipoprotein
T2DM: Type 2 diabetes mellitus

## References

[1] Jannasch F, Kröger J, Schulze MB (2017). Dietary patterns and type 2 diabetes: a systematic literature review and meta-analysis of prospective studies. J Nutr.

[2] Hemmingsen B, Gimenez-Perez G, Mauricio D, Roqué I, Figuls M, Metzendorf MI, Richter B (2017). Diet, physical activity or both for prevention or delay of type 2 diabetes mellitus and its associated complications in people at increased risk of developing type 2 diabetes mellitus. Cochrane Database Syst Rev.

[3] Manios Y, Androutsos O, Lambrinou CP, Cardon G, Lindström J, Annemans L, Mateo-Gallego R, de Sabata MS, Iotova V, Kivelä J, Martinez R, Moreno LA, Rurik I, Schwarz P, Tankova T, Liatis S, Makrilakis K (2018). A school- and community-based intervention to promote healthy lifestyle and prevent type 2 diabetes in vulnerable families across Europe: design and implementation of the Feel4Diabetes-study. Public Health Nutr.

[4] Anastasiou C, Fappa E, Zachari K, Mavrogianni C, Van Stappen V, Kivelä J, Virtanen E, Gonzales-Gil E, Flores-Barrantes P, Nanasi A, Semanova C, Dimova R, Usheva N, Iotova V, Cardong G, Manios Y, Makrilakis K, Feel4Diabetes study group. Development and reliability of questionnaires for the assessment of diet and physical activity behaviors in a multi-country sample in Europe: the Feel4Diabetes Study. BMC Suppl. 2020;20(Suppl 1):135. 10.1186/s12902-019-0469-x PMC7066729 32164677

[5] Lindström J, Tuomilehto J (2003). The diabetes risk score: a practical tool to predict type 2 diabetes risk. Diabetes Care.

[6] Tuomilehto J, Lindström J, Eriksson JG, Valle TT, Hämäläinen H, Ilanne-Parikka P, Keinänen-Kiukaanniemi S, Laakso M, Louheranta A, Rastas M, Salminen V, Uusitupa M (2001). Prevention of type 2 diabetes mellitus by changes in lifestyle among subjects with impaired glucose tolerance. N Engl J Med.

[7] Lindström J, Peltonen M, Eriksson JG, Louheranta A, Fogelholm M, Uusitupa M, Tuomilehto J (2006). High-fibre, low-fat diet predicts long-term weight loss and decreased type 2 diabetes risk: the Finnish diabetes prevention study. Diabetologia.

[8] The Diabetes Prevention Program Research Group (2002). The diabetes prevention program (DPP): description of lifestyle intervention. Diabetes Care.

[9] Galbete C, Kroger J, Jannasch F, Iqbal K, Schwingshackl L, Schwedhelm C, Weikert C, Boeing H, Schulze MB. Nordic diet, Mediterranean diet, and the risk of chronic diseases: the EPIC-Potsdam study. BMC Med. 2018;16(1):99. 10.1186/s12916-018-1082-y PMC6020433 29945632

[10] Becerra-Tomás N, Díaz-López A, Rosique-Esteban N, Ros E, Buil-Cosiales P, Corella D, Estruch R, Fitó M, Serra-Majem L, Arós F, Lamuela-Raventós RM, Fiol M, Santos-Lozano JM, Díez-Espino J, Portoles O, Salas-Salvadó J, PREDIMED Study Investigators (2018). Legume consumption is inversely associated with type 2 diabetes incidence in adults: a prospective assessment from the PREDIMED study. Clin Nutr.

[11] Della Pepa G, Vetrani C, Vitale M, Riccardi G (2018). Wholegrain intake and risk of type 2 diabetes: evidence from epidemiological and intervention studies. Nutrients.

[12] Guasch-Ferré M, Becerra-Tomás N, Ruiz-Canela M, Corella D, Schröder H, Estruch R, Ros E, Arós F, Gómez-Gracia E, Fiol M, Serra-Majem L, Lapetra J, Basora J, Martín-Calvo N, Portoles O, Fitó M, Hu FB, Forga L, Salas-Salvadó J (2017). Total and subtypes of dietary fat intake and risk of type 2 diabetes mellitus in the Prevención con Dieta Mediterránea (PREDIMED) study. Am J Clin Nutr.

[13] Malik VS, Popkin BM, Bray GA, Després JP, Willett WC, Hu FB (2010). Sugar-sweetened beverages and risk of metabolic syndrome and type 2 diabetes: a meta-analysis. Diabetes Care.

[14] Yu E, Hu FB (2018). Dairy products, dairy fatty acids, and the prevention of Cardiometabolic disease: a review of recent evidence. Curr Atheroscler Rep.

[15] Schwingshackl L, Hoffmann G, Lampousi AM, Knüppel S, Iqbal K, Schwedhelm C, Bechthold A, Schlesinger S, Boeing H (2017). Food groups and risk of type 2 diabetes mellitus: a systematic review and meta-analysis of prospective studies. Eur J Epidemiol.

[16] Salas-Salvadó J, Bulló M, Babio N, Martínez-González MÁ, Ibarrola-Jurado N, Basora J, Estruch R, Covas MI, Corella D, Arós F, Ruiz-Gutiérrez V, Ros E, PREDIMED Study Investigators (2011). Reduction in the incidence of type 2 diabetes with the Mediterranean diet: results of the PREDIMED-Reus nutrition intervention randomized trial. Diabetes Care.

[17] Lindström J, Neumann A, Sheppard KE, Gilis-Januszewska A, Greaves CJ, Handke U, Pajunen P, Puhl S, Pölönen A, Rissanen A, Roden M, Stemper T, Telle-Hjellset V, Tuomilehto J, Velickiene D, Schwarz PE, Acosta T, Adler M, AlKerwi A, Barengo N, Barengo R, Boavida JM, Charlesworth K, Christov V, Claussen B, Cos X, Cosson E, Deceukelier S, Dimitrijevic-Sreckovic V, Djordjevic P, Evans P, Felton AM, Fischer M, Gabriel-Sanchez R, Gilis-Januszewska A, Goldfracht M, Gomez JL, Greaves CJ, Hall M, Handke U, Hauner H, Herbst J, Hermanns N, Herrebrugh L, Huber C, Hühmer U, Huttunen J, Jotic A, Kamenov Z, Karadeniz S, Katsilambros N, Khalangot M, Kissimova-Skarbek K, Köhler D, Kopp V, Kronsbein P, Kulzer B, Kyne-Grzebalski D, Lalic K, Lalic N, Landgraf R, Lee-Barkey YH, Liatis S, Lindström J, Makrilakis K, McIntosh C, McKee M, Mesquita AC, Misina D, Muylle F, Neumann A, Paiva AC, Pajunen P, Paulweber B, Peltonen M, Perrenoud L, Pfeiffer A, Pölönen A, Puhl S, Raposo F, Reinehr T, Rissanen A, Robinson C, Roden M, Rothe U, Saaristo T, Scholl J, Schwarz PE, Sheppard KE, Spiers S, Stemper T, Stratmann B, Szendroedi J, Szybinski Z, Tankova T, Telle-Hjellset V, Terry G, Tolks D, Toti F, Tuomilehto J, Undeutsch A, Valadas C, Valensi P, Velickiene D, Vermunt P, Weiss R, Wens J, Yilmaz T (2010). Take action to prevent diabetes--the IMAGE toolkit for the prevention of type 2 diabetes in Europe. Horm Metab Res.

[18] European Public Health Association EUPHA (2017). Healthy and Sustainable Diets for European Countries.

[19] Huseinovic E, Winkvist A, Slimani N, Park MK, Freisling H, Boeing H, Buckland G, Schwingshackl L, Weiderpass E, Rostgaard-Hansen AL, Tjønneland A, Affret A, Boutron-Ruault MC, Fagherazzi G, Katzke V, Kühn T, Naska A, Orfanos P, Trichopoulou A, Pala V, Palli D, Ricceri F, Santucci de Magistris M, Tumino R, Engeset D, Enget T, Skeie G, Barricarte A, Bonet CB, Chirlaque MD, Amiano P, Quirós JR, Sánchez MJ, Dias JA, Drake I, Wennberg M, Boer J, Ocké MC, Verschuren W, Lassale C, Perez-Cornago A, Riboli E, Ward H, Forslund HB (2016). Meal patterns across ten European countries - results from the European Prospective Investigation into Cancer and Nutrition (EPIC) calibration study. Public Health Nutr.

[20] Lassale C, Gunter MJ, Romaguera D, Peelen LM, Van der Schouw YT, Beulens JW, Freisling H, Muller DC, Ferrari P, Huybrechts I, Fagherazzi G, Boutron-Ruault MC, Affret A, Overvad K, Dahm CC, Olsen A, Roswall N, Tsilidis KK, Katzke VA, Kühn T, Buijsse B, Quirós JR, Sánchez-Cantalejo E, Etxezarreta N, Huerta JM, Barricarte A, Bonet C, Khaw KT, Key TJ, Trichopoulou A, Bamia C, Lagiou P, Palli D, Agnoli C, Tumino R, Fasanelli F, Panico S, Bueno-de-Mesquita HB, Boer JM, Sonestedt E, Nilsson LM, Renström F, Weiderpass E, Skeie G, Lund E, Moons KG, Riboli E, Tzoulaki I (2016). Diet Quality Scores and Prediction of All-Cause, Cardiovascular and Cancer Mortality in a Pan-European Cohort Study. PLoS One.

