# Supplementary material for: Feel4Diabetes healthy diet score: development and evaluation of clinical validity
Source: BMC Endocr Disord. 2020 May 6;20(Suppl 2):46. doi: 10.1186/s12902-020-0521-x (PMC7201941; doi:10.1186/s12902-020-0521-x)
Supplement: Supplementary file 1 — Additional file 1. Feel4Diabetes dietary questions used in the development of the Healthy Diet score. [file 12902_2020_521_MOESM1_ESM.docx]

**Additional file 1**. Feel4Diabetes dietary questions used in the development of the Healthy Diet score

| Component | Questions included |
| --- | --- |
| 1. Breakfast | How often do you consume main meals on weekdays/weekend days?  Breakfast   - never - less than 1 time/week - 1-2 times/week - 3-4 times/week - 5-6 times/week - daily |
| 2. Vegetables | How many servings of raw or cooked vegetables do you eat?  One serving is half a cup of vegetables or at the size of a tennis ball tomato, broccoli or leafy vegetables.   - Less than one serving per week - 1-2 servings per week - 3-4 servings per week - 5-6 servings per week - 1-2 servings per day - 3-4 servings per day - 5 or more servings per day   How many times per week do you eat legumes (e.g. lentils, beans, peas)? ____  What is your average portion size? Please consider the legumes cooked and drained, without soup.   - Less than half a cup - Half a cup (100 g) - 1 cup - 1.5 cup - 2 cups - 2.5 cups - 3 cups - 8. More than 3 cups |
| 3. Fruit and berries | How many servings of fruits or berries do you eat?  For fresh or frozen fruits, one serving is a tennis ball (medium) sized fruit (e.g. apple) or two small fruits (e.g. apricots) or half a cup of chopped fruit or berries. For canned fruits, one serving is half a cup of canned fruits. For dried fruits, one serving is a quarter of a cup (62.5 mL) of dry fruits and berries.   - Less than one serving per week - 1-2 servings per week - 3-4 servings per week - 5-6 servings per week - 1-2 servings per day - 3-4 servings per day - 5 or more servings per day |
| 4. Sugary drinks | How much of the following beverages do you drink per week?  Mark 0, if you don´t drink the following beverages or if you drink on average less than one serving per week.  1. _____ glass(es) of water (1 glass=250 mL)  2. _____ cup(s) of tea (1 cup=250 mL)  3. _____ cup(s) of coffee (1 cup=250 mL)  4. _____ glass(es) of soft drink with sugar (1 glass = 250 mL)  5. _____ glass(es) of soft drink without sugar, e.g. Coca Cola Light (1 glass = 250 mL)  6. _____ glass(es) of fruit juice freshly squeezed or prepacked without sugar (1 glass = 250 mL)  7. _____ glass(es) of juice containing sugar (1 glass =250 mL)  8. _____ glass(es) of beer/cider (1 beer glass = 330 mL)  9. _____ glass(es) of wine (1 wine glass = 125 mL) |
| 5. Whole-grain | How much bread and other cereals do you eat per day?  A roll (60 g) equals 2 slices (30 g) of bread. Mark 0 if you eat on average less than one serving per day.  1.______ slice(s) of whole grain bread  2.______ slice(s) of non-whole grain (i.e. white) bread  3.______ cup(s) of porridge (e.g. rye, oat or wheat flake porridge)  4.______ cup(s) of whole grain breakfast cereals, muesli  5.______ cup(s) of non-whole grain breakfast cereals (e.g. corn flakes or rice crispies) |

| 6. Nuts and seeds | How many servings of nuts or seeds do you eat?  One serving is about 2 tablespoons or 30 g   - Less than one serving per week - 1-2 servings per week - 3-4 servings per week - 5-6 servings per week - 1-2 servings per day - 3-4 servings per day - 5 or more servings per day |
| --- | --- |
| 7. Low-fat dairy | How many servings of milk and milk products (e.g. yogurt) do you consume per day?  One serving is half a cup (120 mL). Count also milk in tea, coffee or with cereal. Do not count cheese. Mark 0 if you eat on average less than one serving per day.  1.______ servings of low-fat or fat-free milk and milk products (< 2 % fat)  2.______ servings of full fat milk and milk products (> 2 % fat) |
| 8. Oils and fats | What kind of fat spread do you usually use with your bread?  Choose only one option.   - Vegetable oil (olive, rapeseed etc.) - Soft margarine with 70-80% fat - Reduced-fat margarine 28-60% fat - Butter-vegetable oil mixture - Butter - I do not usually have fat spread on bread   What of the following fats and oils do you use daily? Think about fats and oils used with vegetables (raw or boiled) and/or in cooked dishes (added during or after cooking).  Choose one or more options.   - Olive or rapeseed oil - Other vegetable oil (e.g. sunflower oil) - Margarine - Butter - Mayonnaise, French dressing etc. - I do not use any of these fats daily |
| 9. Red meat | How many times per week do you eat red meat (e.g. pork, beef, veal, lamb) or processed meat (e.g. hamburger or sausages)? ______ time(s) per week  What is your average portion size?   - Less than 1 pack of cards - 1 pack of cards (100-120 g) - 1.5 pack of cards - 2 packs of cards - 2.5 packs of cards - 3 packs of cards - 3.5 packs of cards - 4 packs of cards - more than 4 packs of cards |
| 10.Sweet snacks | How many servings of sweets, biscuits, ice cream, cakes, pastries times do you eat? (One serving is 1 small chocolate bar (40 g) or half a cup of sweets, cookies or 1 scoop of ice cream)  One or less than one serving per week   - 2 servings per week - 3-4 servings per week - 5-6 servings per week - 1-2 servings per day - 3-4 servings per day - 5 or more servings per day |
| 11. Salty snacks | How many servings of salty snacks/fast food do you eat?  (One serving is: 1 small bag of chips, 1 slice of pizza, 1 cheese pie or other dough-based snacks)   - One or less than one serving per week - 2 servings per week - 3-4 servings per week - 5-6 servings per week - 1-2 servings per day - 3-4 servings per day - 5 or more servings per day |
| 12. Family meals | How often do you have the following meals with others, with family, friends or colleagues?  1. Breakfast/2. Lunch/3. Dinner   - never - less than 1 time/week - 1-2 times/week - 3-4 times/week - 5-6 times/week - daily |
